# All Sensilla on the Body of the Smallest Beetle *Scydosella musawasensis* (Coleoptera: Ptiliidae) and Their Implications for the Evolution of the External Sensory Apparatus During Miniaturization

**DOI:** 10.3390/insects17100985

**Published:** 2026-09-24

**Authors:** Kseniia T. Abu Diiak, Alexey A. Polilov

**Affiliations:** Department of Entomology, Faculty of Biology, Lomonosov Moscow State University, Leninskie Gory 1-12, Moscow 119234, Russia; polilov@gmail.com

**Keywords:** miniaturization, *Scydosella*, sensilla, sensory modalities, sensory system, Ptiliidae

## Abstract

Extreme miniaturization may impose severe constraints on the organization of insect sensory systems, yet the extent to which sensory diversity can be reduced while retaining essential functions remains poorly understood. Here we characterize the sensilla distributed across the whole body surface of the adult minute featherwing beetle *Scydosella musawasensis*, the smallest known species of free-living insect. We identify four principal types of sensilla and four subtypes of trichoid sensilla, with 851–883 sensilla distributed across the body surface excluding the antennae, studied earlier. Despite this quantitative reduction compared to other Staphylinoidea, the beetle retains the sensory modalities required for interacting with its environment. Mechanosensory trichoid sensilla dominate the body surface, whereas campaniform and thorn-like trichoid sensilla are concentrated near appendage articulations and probably contribute to proprioception. Basiconic sensilla on the maxillary palps are consistent with contact chemoreception, whereas coeloconic sensilla on the head may perform thermo- and hygroreception. Thus, the distribution of sensilla types and modalities is similar to those of other insects. The sensillar complement and distribution are remarkably similar between males and females and stable between individuals. Together with observations of the antennae, these findings confirm that extreme miniaturization primarily reduces the number of sensory units while preserving the diversity and organization of the major sensory modalities.

## Simple Summary

Insects need sensory organs to detect their surroundings, find food, move through their environment and reproduce. However, very small insects have limited space for these organs, and it is not known how much their sensory system can be reduced while still supporting normal life. We studied the sensory hairs and other small sensory structures covering the body of *Scydosella musawasensis*, the smallest known free-living insect. We found that this tiny beetle has far fewer sensory structures than larger insects, but it still retains different kinds of structures that can detect touch, movement, chemicals and environmental conditions. These structures are also arranged in a highly consistent pattern, with very little difference between males and females. Our results show that extreme reduction in body size mainly decreases the number of sensory structures rather than removing important types of sensory organs. This study helps explain how insects can maintain essential functions despite severe limits on body size and provides insight into the basic requirements for a functioning sensory system.

## 1. Introduction

Sensory organs and other sensory structures are fundamental to insect survival, providing essential input about external conditions and internal states. This information guides key behaviors such as locating food, identifying mates, spatial orientation, and coordinating movement [1]. Among these organs, sensilla are particularly important. These specialized cuticular structures, each typically innervated by at least one bipolar neuron and accompanied by auxiliary cells, function as the interface between the insect and its environment. Distributed across the entire body, they are found in highest concentrations on the antennae, mouthparts, legs, wings, and genitalia [2]. Functionally, sensilla are diverse, serving as mechano-, chemo-, thermo-, and hygroreceptors [3].

Several classification systems have been developed to organize this diversity based on structure. Snodgrass [4] emphasized external morphology, distinguishing between hair-like, plate-like, campaniform, and scolopophorous types of sensilla. The hair-like category alone encompasses several forms, including sensilla trichodea, chaetica, basiconica, coeloconica, ampullacea, and squamiformia. Later, Altner and Prillinger [5] introduced a classification based on cuticular pores, separating sensilla into the aporous, uniporous, and multiporous (further divided into single- or double-walled) categories. These structural differences correlate with function: aporous sensilla are typically mechano-, hygro-, or thermoreceptors; uniporous sensilla often function as mechanoreceptors or contact chemoreceptors; and multiporous sensilla commonly house chemo-, thermo-, and hygroreceptive cells. An additional distinction, proposed by Slifer [6], separates chemoreceptive sensilla into thin-walled (olfactory) and thick-walled (contact chemoreceptors) types. Elgar and Freelance [7] classify antennal sensilla based on their primary function, such as chemosensory sensilla, mechanoreceptors, hygroreceptors and thermoreceptors.

Miniaturization represents a major evolutionary trajectory in insects, often resulting in body sizes that approach the limits seen in unicellular organisms. This dramatic reduction in scale necessitates profound reorganization across all organ systems, often down to the cellular level [8,9,10]. The sensory apparatus is not exempt from these pressures, and its study in miniature insects offers unique insights into structural optimization and the constraints imposed by extreme size reduction.

While the sensory structures of many insect species have been thoroughly documented [3,11,12,13,14,15], systematic investigations into how these systems scale with body size are relatively recent and remain scarce. Existing research has largely concentrated on the eyes and antennae. These studies reveal a clear link between body size and the architecture of sensory organs, both across different species [16,17,18] and within a single species [19,20,21]. Recent reviews have synthesized findings on the scaling of insect eyes [22] and sensilla [23]. A recurring theme is the reduction in the number of functional units—whether ommatidia or sensilla—as body size decreases. For instance, the minute parasitoid wasp *Megaphragma viggianii* possesses far fewer ommatidia than its larger relatives [24] and also exhibits a reduced number of antennal and mouthpart sensilla [25,26,27]. Investigations into the antennae of the featherwing beetle *Scydosella musawasensis* have demonstrated a similar trend: smaller individuals bear fewer sensilla, although the diversity of sensillar types and their individual dimensions remain consistent [28]. Beyond numerical reduction, miniaturization can also lead to structural simplification, as seen in *M. viggianii*, where certain placoid sensilla lack auxiliary cells [27].

Ranking as the smallest known free-living insect, *Scydosella musawasensis* reaches a body length of merely 0.3 mm [9,29]. As a member of the Ptiliidae family, it showcases a suite of adaptations to its minute size [30]. Its diminutive stature places it near the theoretical lower limits for insect body size, making it an invaluable model for exploring the fundamental constraints on the structure of sensory systems. To thrive, *S. musawasensis* must still navigate its environment, locate food sources (presumably fungal spores), and find mates—tasks that demand a fully operational sensory system.

Although interest in the effects of miniaturization on insect sensory systems is growing, comprehensive data on the sensory structures of the smallest insects remain limited. A more complete understanding of the principles and boundaries of sensory system miniaturization requires detailed information on the structure and distribution of these structures. While the antennal sensilla of *S. musawasensis* have been examined earlier, offering initial evidence of scaling effects [28], a more in-depth morphological analysis is necessary to fully characterize the types, arrangement, and potential functional roles of all sensilla in adults of this species. This study aims to expand our knowledge of the miniaturization of sensory structures by providing a detailed investigation of the sensilla on the whole body surface of *S. musawasensis*.

## 2. Materials and Methods

We examined 26 adult males and 27 adult females of *Scydosella musawasensis* Hall, 1999 (Coleoptera: Ptiliidae) (Figure 1). The specimens were collected in Chicaque Natural Park, Columbia; some of them were examined and measured earlier [29]. The collected material was fixed in FAE (formaldehyde—acetic acid—ethanol) and stored in 70% ethanol.

For scanning electron microscopy (SEM), dried specimens were mounted on microscopy holders using conductive tape and coated with a 20 nm gold layer using a Cressington-208HR Ion Coater (Cressington Scientific Instruments Ltd., Watford, UK). Images of all body parts and individual sensilla were captured using Jeol JSM-6380 (JEOL Ltd., Tokyo, Japan) and FEI Quattro S (Thermo Fisher Scientific Inc., Waltham, MA, USA) scanning electron microscopes. To visualize the concealed medial sides of mouthparts, heads of multiple specimens were bisected through the mouthparts using razor blades. These head sections were then placed in water droplets on glass fragments attached to scanning electron microscopy holders. Following drying, the same coating procedure was applied to these preparations.

Measurements of sensilla length and basal width were obtained from image series using ImageJ 1.54p software [31]. At least 10 sensilla of each type were measured. For trichoid, basiconic, and coeloconic sensilla, we measured their length from the base to the tip, excluding the socket, as well as their width at the base (i.e., the most proximal visible part). For campaniform sensilla, we measured the diameter of the cuticular dome. The resulting data were utilized to compute sensilla dimensions, and the minimum and maximum values for these structures.

## 3. Results

### 3.1. Types of Sensilla

Four types and four subtypes of sensilla were identified on the body surface of *S. musawasensis* (Figure 2) excluding antennae, which have been studied earlier [28].

Trichoid sensilla (Figure 2A–E) are elongated, hair-like structures without visible sockets. Their width gradually decreases from the base to the tip. The tips may be rounded or pointed, and some trichoid sensilla exhibit slight longitudinal striation; however, no pores are visible on their surface. Trichoid sensilla can be divided into four subtypes. Curved trichoid sensilla (Figure 2A,B, cts) constitute the vast majority of sensilla on the body surface of *S. musawasensis*. These sensilla usually stand upright and have a curved distal portion with rounded tips. Their length is 5–15 µm; their width is 0.6–1 µm. Porous trichoid sensilla (Figure 3D, tsp) are morphologically similar to curved trichoid sensilla, but have pores on their tips. Pointed trichoid sensilla (Figure 2C, pts) have straight shafts and pointed tips. Unlike curved trichoid sensilla, they lie flat against the cuticular surface and never stand upright. The length of these structures is 10–15 µm; their width is 0.6–1 µm. Thorn-like trichoid sensilla (Figure 2D,E, tts) are short (1–5 µm in length) and relatively wide (0.7–1.5 µm in width) structures with a conical shape and rounded tips.

Coeloconic sensilla (Figure 2F, cos) are very small (less than 0.5 µm in both length and width), cone-shaped structures surrounded by a cuticular ring. No grooves or pores are visible on their surface. These sensilla are present only on the posterior region of the head capsule.

Campaniform sensilla (Figure 2G, cfs) appear as small cuticular domes with smooth surfaces lacking grooves or pores. Their margins gradually merge with the surrounding cuticle. Their diameter is 1–2.5 µm. These sensilla are located at the bases of the wings and on leg segments near the articulations.

Basiconic sensilla (Figure 2H,I, bcs) are very thick (2–3 µm wide, and 6–9 µm long) sensilla located on the third segment of each maxillary palp. They have rounded apices bearing a characteristic group of pores (Figure 2I). These sensilla lack sockets, and their bases gradually merge with the surrounding cuticle.

We also noticed an unknown structure positioned near the tip of the third maxillary palpomere (Figure 3F, smp). It is 2–3 µm in length and has deep longitudinal grooves separating it into several compartments. Although it was described as a digitiform sensillum by Sörensson [32], it remains unclear whether it is a sensillum or not.

### 3.2. Distribution of Sensilla

The head capsule (excluding the mouthparts and antennae) bears only curved trichoid and coeloconic sensilla on its surface (Figure 3A). The ventral surface of the head has four (rarely three) curved trichoid sensilla arranged in a transverse row proximal to the mouthparts (Figure 3B). The dorsal surface bears 16 curved trichoid sensilla located between the compound eyes. Each eye has seven sensilla of this type situated between the ommatidia. The posterior part of the head capsule bears six coeloconic sensilla on its lateral surfaces, with three sensilla on each side. The head capsule has 40 sensilla in total.

The mouthparts bear curved trichoid, porous trichoid, thorn-like trichoid, and basiconic sensilla (Figure 3B–D and Figure 4). The clypeus bears 17 distally inclined curved trichoid sensilla (Figure 3A and Figure 4A). Each mandible bears two thorn-like trichoid sensilla on its lateral surface (Figure 3E and Figure 4B). The maxilla consists of the cardo, stipes, galea, lacinia, and maxillary palp. The cardo bears two curved trichoid sensilla on its ventral surface and a group of four thorn-like trichoid sensilla on its medial surface (Figure 3B and Figure 4C). The stipes bears three curved trichoid sensilla on its lateral surface. The lacinia has one thorn-like trichoid sensillum on its ventral surface, whereas the galea bears six curved trichoid sensilla on its medial surface. The maxillary palp consists of three segments. The first segment lacks sensilla, whereas the second bears a single curved trichoid sensillum on its lateral surface. The third segment has the greatest diversity of sensilla, bearing five curved trichoid sensilla (two on the lateral surface and three on the medial surface) and one basiconic sensillum at its apex (Figure 3B,C and Figure 4C,D). The labium consists of the prementum and postmentum, the latter comprising the mentum and submentum. The submentum bears seven curved trichoid sensilla on its ventral surface (Figure 3B and Figure 4E). The base of the mentum bears two curved trichoid sensilla, whereas its distal part bears a pair of labial palps. Each labial palp bears one curved trichoid sensillum and one porous trichoid sensillum (Figure 3D). The mouthparts have 80 sensilla in total.

The thorax bears curved trichoid and thorn-like trichoid sensilla (Figure 5). The ventral surface of the prothorax has relatively few sensilla, consisting only of a transverse row of six curved trichoid sensilla near the anterior margin (Figure 5A). The dorsal surface bears 30 curved trichoid sensilla arranged in six longitudinal rows (Figure 5B). The ventral surface of the mesothorax bears a medial group of four curved trichoid sensilla (Figure 5C). The dorsal surface of the mesothorax bears only four sensilla, all located on the scutellum: two thorn-like trichoid sensilla on the anterior part and two curved trichoid sensilla on the posterior part (Figure 5D). The dorsal surface of the metathorax bears 12 thorn-like trichoid sensilla, arranged as two groups of six located near the base of each wing (Figure 6A). The ventral surface of the metathorax bears a total of 14 curved trichoid sensilla: a row of six near the anterior margin, a similar row of six near the posterior margin, and two sensilla located between these rows (Figure 5C). The thorax has 70 sensilla in total.

The elytra (fore wings) and hind wings bear curved trichoid, pointed trichoid, and campaniform sensilla (Figure 6). The dorsal surface of each elytron bears 75–85 curved and pointed trichoid sensilla (Figure 6B), and is one of the few exceptions with an unstable number of sensilla. Curved trichoid sensilla are concentrated in the anterior and posterior regions of the elytra, whereas pointed trichoid sensilla are more abundant in the middle region. The ventral surface of each elytron bears a row of six campaniform sensilla extending along the elytral suture (Figure 6C). The base of each hind wing bears one campaniform sensillum (Figure 6A). The middle region of the wing bears a row of eight curved trichoid sensilla along its posterior margin, which continues into a row of long serrated setae used in flight (Figure 6D). The same region also bears a row of three long setae along the anterior margin. Similar long setae are present on the distal part of the wing. Each wing has nine sensilla in total.

The legs bear curved trichoid, thorn-like trichoid, and campaniform sensilla (Figure 7 and Figure 8). The procoxae and mesocoxae each bear two curved trichoid sensilla, one on the ventral surface and one on the dorsal surface. The metacoxae bear five curved trichoid sensilla on the ventral surface (Figure 7A and Figure 8). The coxae of all legs also have a single campaniform sensillum positioned distally. The trochanter of each leg bears two curved trichoid sensilla on its ventral surface. These sensilla are positioned closer to the distal end of each leg segment in the forelegs and midlegs but are shifted proximally in the hindlegs. The profemora and mesofemora each bear seven curved trichoid sensilla on the ventral surface, whereas the metafemora bear five (Figure 7B and Figure 8). The proximal part of all femora bears a campaniform sensillum on the ventral surface. The dorsal surface of all femora lacks sensilla. The tibiae bear 13 curved trichoid sensilla on the forelegs, 18 on the midlegs, and 26 on the hindlegs (Figure 7C and Figure 8). These sensilla are most densely distributed on the distal part of the tibiae, whereas the dorsal surfaces are largely devoid of sensilla. The protibiae and mesotibiae each bear seven thorn-like trichoid sensilla on their distal part, forming a ventral group on the forelegs and a complete ring on the midlegs. The metatibiae bear two thorn-like trichoid sensilla on their distal part. In the forelegs and midlegs, the first tarsomere bears four curved trichoid sensilla and the second bears three. In the hindlegs, the first tarsomere bears three curved trichoid sensilla, whereas the second bears two (Figure 7D and Figure 8). Each foreleg has 40 sensilla in total, each midleg has 45, and each hindleg has 47.

The abdomen bears curved trichoid and thorn-like trichoid sensilla (Figure 9). Abdominal tergites 1–8 each bear a single row of curved trichoid sensilla; tergite 9 bears two distinct rows, and the sensilla on tergite 10 are not arranged into rows (Figure 9A). Unlike other body parts, the rows on the tergites have a varying number of sensilla in different individuals. These sensilla are distributed as follows: tergite (t) 1–8, t2–11, t3–12, t4–12 to 13, t5–13 to 15, t6–13 to 15, t7–6 to 9, t8–6 to 7, t9–15 to 16 and 7 to 8 (two rows), t10–30. Sternites 2–6 each bear a row of curved trichoid sensilla on their distal margin, whereas sternites 7–8 are fused, and the sensilla on their surface are not arranged into rows (Figure 9B). The numbers of sensilla are distributed as follows: sternite (s) 2–4, s3–4, s4–8, s5–10, s6–10, s7+8–44 (males) or 45 (females). Each pleurite of the abdominal segment 2 bears a single thorn-like trichoid sensillum, those of segments 3–5 each bear two thorn-like trichoid sensilla, whereas the pleurites of segments 6–7 each bear four curved trichoid sensilla. The abdomen has 229–241 sensilla in total.

The distribution of sensilla is identical in males and females, besides the sensilla located on the ventral surface of the pygidia (Figure 9C,D). The complement of sensilla is generally stable, with only rare aberrations observed in some individuals. The total number of sensilla on the whole body without antennae is 851–883.

## 4. Discussion

### 4.1. Structure and Possible Functions of the Sensilla

In *Scydosella musawasensis*, the diversity of sensilla on the body surface excluding the antennae is relatively low and comprises four principal morphological types with four trichoid subtypes. Nevertheless, these sensilla most likely perform the full range of sensory functions required for survival, including mechanoreception, chemoreception, and the perception of environmental parameters such as humidity or temperature. The antennae have the greatest diversity of sensilla among all body parts: they bear chaetoid sensilla, four subtypes of trichoid sensilla, and two subtypes of both styloconic and basiconic sensilla [28].

Trichoid sensilla constitute the vast majority of the sensilla on the body surface of *S. musawasensis* (Figure 10). Their elongated hair-like shape, smooth aporous cuticle, and absence of terminal pores (except one subtype, porous trichoid sensilla) indicate that they most likely function as mechanoreceptors. Similar sensilla have been interpreted as tactile mechanoreceptors in numerous beetles and other insects, where they respond to displacement of the hair shaft [33]. The abundance of curved trichoid sensilla on the distal parts of the legs is consistent with a role in substrate exploration and locomotion, whereas those located on the mouthparts probably assist during feeding. The pointed trichoid sensilla differ from the curved subtype in both morphology and orientation. Their straight shafts lie closely appressed to the cuticle, suggesting that they do not function as mechanoreceptors and are most likely non-innervated hairs. Thorn-like trichoid sensilla are considerably shorter and stouter than the other trichoid subtypes. They are mostly located between segment articulations, which suggests that they function as proprioceptors, perceiving the position and movement of the segments relative to each other. Porous trichoid sensilla located on labial palps have terminal pores and likely perform both mechanosensory and gustatory functions. One of the four subtypes of trichoid sensilla on antennae has pores on its surface and likely functions as an olfactory chemoreceptor, while the other antennal trichoid sensilla have no pores and are probably mechanoreceptors [28]. There is also a possibility that at least some trichoid sensilla are non-innervated hairs. Determining this would require studying their ultrastructure and is a topic for future research.

Chaetoid sensilla are only located on flagellomeres 7 and 8. They have no pores and are described as typical mechanoreceptors similar to trichoid sensilla [28].

The basiconic sensilla located on the terminal maxillary palpomere and antennal flagellomere 8 [28] bear numerous cuticular pores. According to the classification of Altner and Prillinger [5], these are multiporous sensilla, which are generally associated with chemoreception. Their position at the apex of the maxillary palp strongly suggests that they function as contact chemoreceptors involved in food evaluation. Maxillary palpal basiconic sensilla have gustatory or olfactory functions in different insects [34,35], allowing discrimination of potential food sources before ingestion. Since *S. musawasensis* presumably feeds on fungal material, these sensilla may enable the recognition of suitable fungal substrates through chemical cues. The antennal basiconic sensilla likely perform olfactory functions [28] and have a different pore distribution: the pores cover their whole surface unlike the basiconic sensilla on the maxillary palps which only have pores on their tips.

Styloconic sensilla are only located on antennal flagellomeres 7 and 8 and are supposed to be thermo- and hygroreceptors [28].

Campaniform sensilla occur exclusively near articulations of the legs and wings. Their dome-shaped morphology corresponds well to the typical structure of campaniform sensilla described throughout insects [36,37,38]. These sensilla are widely recognized as proprioceptors that detect cuticular deformation generated by movements or mechanical loading of appendages [39]. Their position at articulations indicates that they provide sensory feedback important for coordinating locomotion and flight.

Coeloconic sensilla are restricted to the posterior region of the head capsule. Their recessed position within a cuticular cavity and lack of visible pores suggest that they are unlikely to function as contact chemoreceptors. In insects, coeloconic sensilla commonly serve as thermo- and hygroreceptors [40], although some also detect volatile chemical compounds. Because these sensilla are located away from the mouthparts and appendages, they most likely monitor ambient environmental conditions rather than participate in tactile perception.

Overall, the sensory equipment of *S. musawasensis* appears highly simplified quantitatively but not qualitatively. All major functional types of sensilla remain represented (Figure 10) despite the extreme reduction in body size, confirming that miniaturization primarily decreases the number of sensory units while preserving essential sensory modalities. The distribution of sensilla across body parts is generally similar to that of other insects [2,3]. Mechanoreceptors cover the whole body surface (curved trichoid sensilla), being especially abundant on legs, near segment articulations and wing bases (thorn-like trichoid and campaniform sensilla). Contact chemoreceptors are present on mouthparts (basiconic and porous trichoid sensilla), while olfactory chemoreceptors as well as thermo- and hygroreceptors (styloconic sensilla) are concentrated on the antennae [28].

### 4.2. Sexual Dimorphism and Stability of the Sensillar Pattern

The structure, diversity, distribution, and dimensions of sensilla in *S. musawasensis* show virtually no sexual dimorphism. Apart from the sensilla located on the ventral surface of the pygidium, males and females possess identical complements of sensilla. The antennal sensilla of *S. musawasensis* have been previously shown to have no sexual dimorphism either [28]. The arrangement of sensilla on the body surface is remarkably stable among individuals, with only minor random deviations affecting the number of sensilla on several body regions. The sensilla distribution is the most variable on elytra and abdominal tergites, where the numbers often slightly fluctuate among different individuals.

The absence of sexual dimorphism contrasts with numerous other beetles in which specialized sensilla are associated with courtship or mate recognition. For example, males of at least several Leiodidae species possess modified adhesive tarsal setae that facilitate attachment to females during copulation [41]. In contrast, *S. musawasensis* apparently lacks such specialized sensory adaptations, suggesting that adults of both sexes perform largely similar sensory tasks throughout their life.

The lack of sexual differences is also consistent with observations in other miniature insects. The parasitoid wasp *Megaphragma viggianii* likewise exhibits no sexual dimorphism in the structure or distribution of mouthpart sensilla [25], although its antennae are sexually dimorphic [26]. This pattern suggests that miniaturization may constrain the evolution of sexually specialized peripheral sensory systems unless they provide a substantial selective advantage.

Certain chaetotaxy characters, particularly those of the mouthparts and individual sternites, are already actively used for species identification [42,43,44]. The stable set of sensilla on other parts of the body suggests that the range of diagnostic characters associated with them could be considerably expanded.

### 4.3. Comparison with Other Staphylinoidea

The sensory system of *S. musawasensis* largely retains the typical complement of sensilla (chaetoid, trichoid, campaniform, coeloconic, basiconic, and styloconic sensilla) characteristic of other representatives of the superfamily Staphylinoidea [45,46,47,48]. The distribution of sensilla also remains highly conserved. Trichoid sensilla remain distributed over the whole body surface, campaniform sensilla are retained near articulations, and chemoreceptive basiconic sensilla remain confined to the maxillary palps. Such conservation indicates that functional requirements impose strong constraints on sensory system organization even at extremely small body sizes.

However, compared with larger representatives of related groups, which typically possess several thousand or more sensilla [45,46,47,48], *S. musawasensis* has a substantially smaller total number of sensilla (1113–1145 including those on the antennae) and exhibits a highly stereotyped pattern of sensillar distribution across the body surface. Similar patterns have been reported in the miniature parasitoid wasp *Megaphragma viggianii* (Hymenoptera: Trichogrammatidae), which also possesses far fewer sensilla than larger hymenopterans [25]. This similarity may reflect general principles underlying the miniaturization of insect sensory systems.

Taken together, these findings support the hypothesis that extreme miniaturization primarily affects the quantitative organization of insect sensory systems rather than their qualitative composition. *Scydosella musawasensis* preserves the full set of major sensillar types characteristic of Staphylinoidea while minimizing their number to what is probably close to the lower functional limit. These results contribute to our understanding of the scaling of insect sensory systems and help define the minimum sensory equipment capable of supporting normal behavior in the smallest known species of free-living insect.

## Figures and Tables

**Figure 1:**
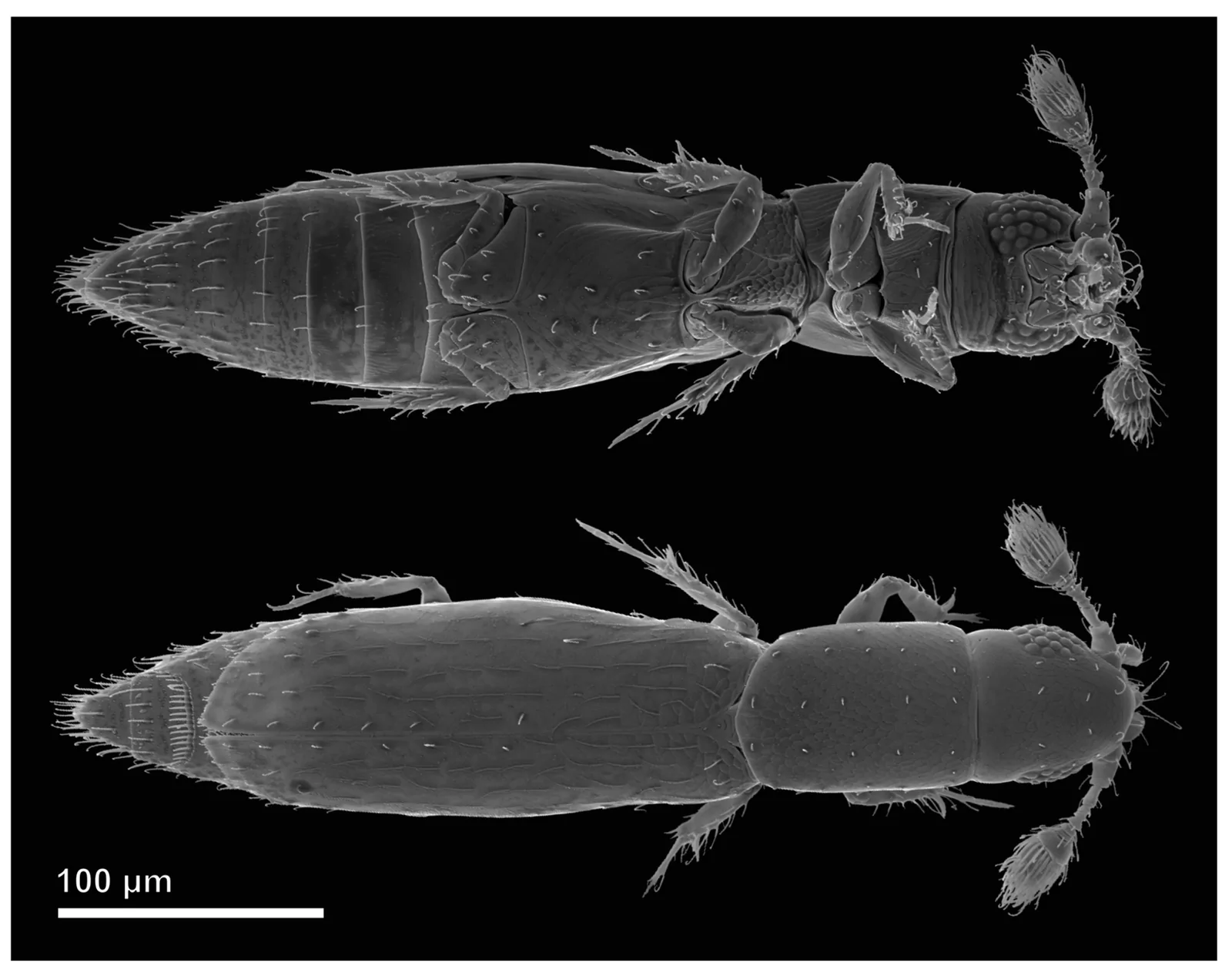
Male *S. musawasensis* in ventral and dorsal view (SEM).

**Figure 2:**
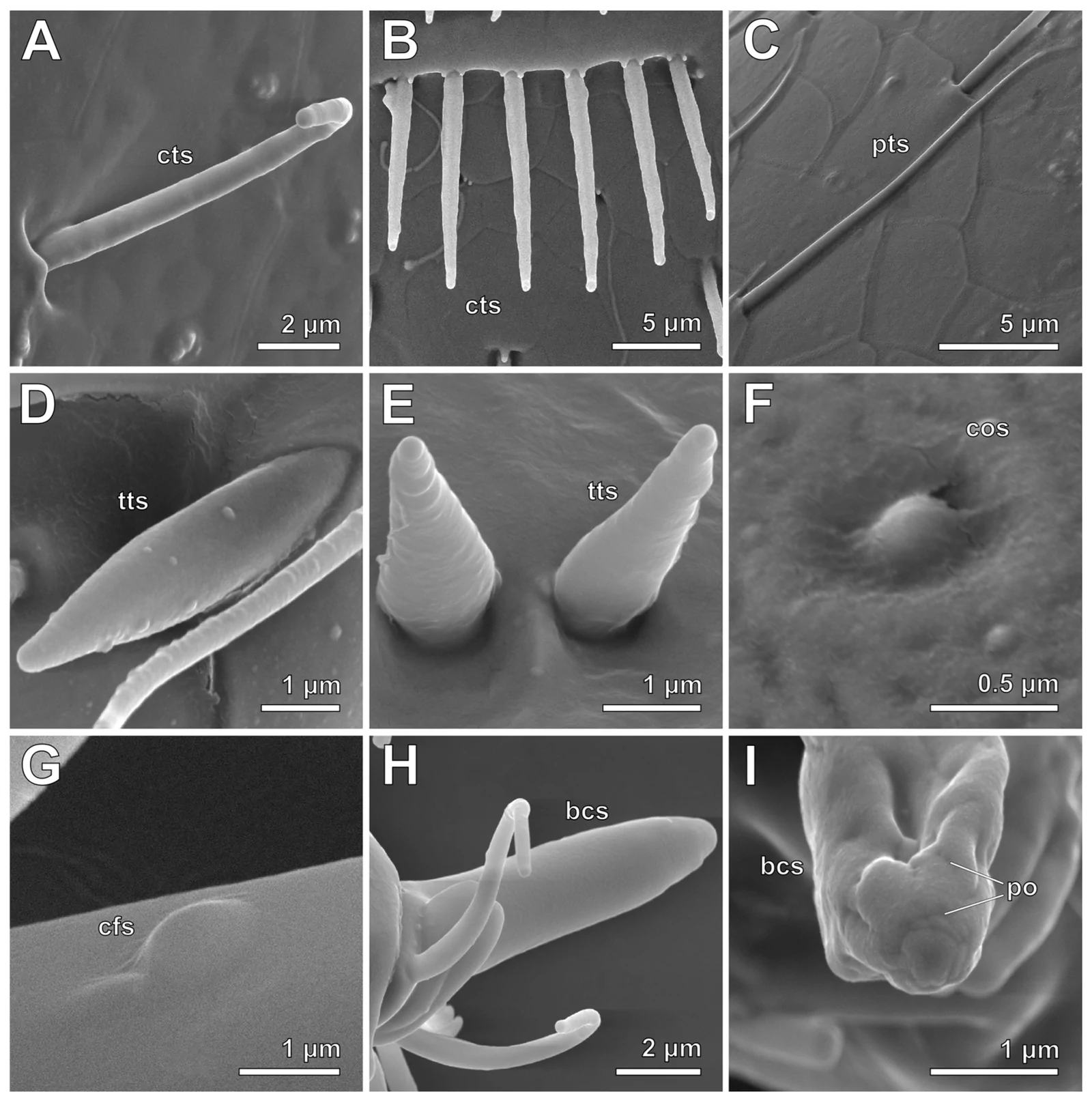
Types of sensilla in *S. musawasensis* (SEM). (**A**). Curved trichoid sensillum on dorsal surface of elytron. (**B**). Row of curved trichoid sensilla on the abdominal tergite 7. (**C**). Pointed trichoid sensillum on dorsal surface of elytron. (**D**). Thorn-like trichoid sensillum on distal part of mesotibia. (**E**). Thorn-like trichoid sensilla on lateral surface of abdomen. (**F**). Coeloconic sensillum on posterior region of head capsule. (**G**). Campaniform sensillum on proximal part of metafemur. (**H**). Basiconic sensillum on maxillary palpomere 3. (**I**). Pores on tip of basiconic sensillum. Abbreviations: bcs—basiconic sensilla; cfs—campaniform sensilla; cos—coeloconic sensilla; cts—curved trichoid sensilla; po—pores; pts—pointed trichoid sensilla; tts—thorn-like trichoid sensilla.

**Figure 3:**
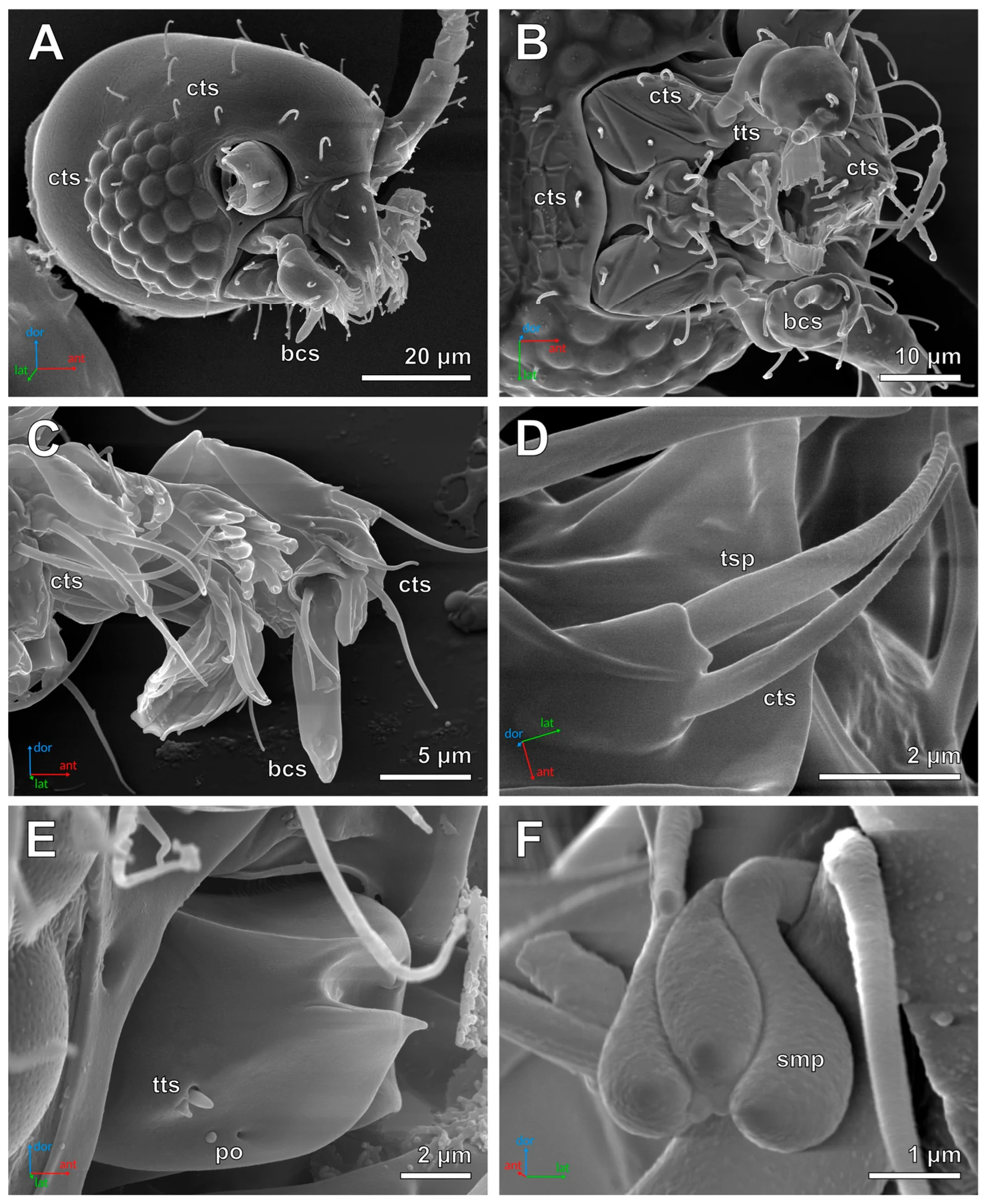
Head structures of *S. musawasensis* (SEM). (**A**). Lateral view of head, female. (**B**). Ventral view of mouthparts, male. (**C**). Medial view of mouthparts, male. (**D**). Labial palp, male. (**E**). Mandible, female. (**F**). Structure on the third maxillary palpomere, male. Abbreviations: bcs—basiconic sensilla; cts—curved trichoid sensilla; po—pores; smp—structure on the third maxillary palpomere; tsp—porous trichoid sensilla; tts—thorn-like trichoid sensilla; ant—anterior direction; dor—dorsal direction; lat—lateral direction.

**Figure 4:**
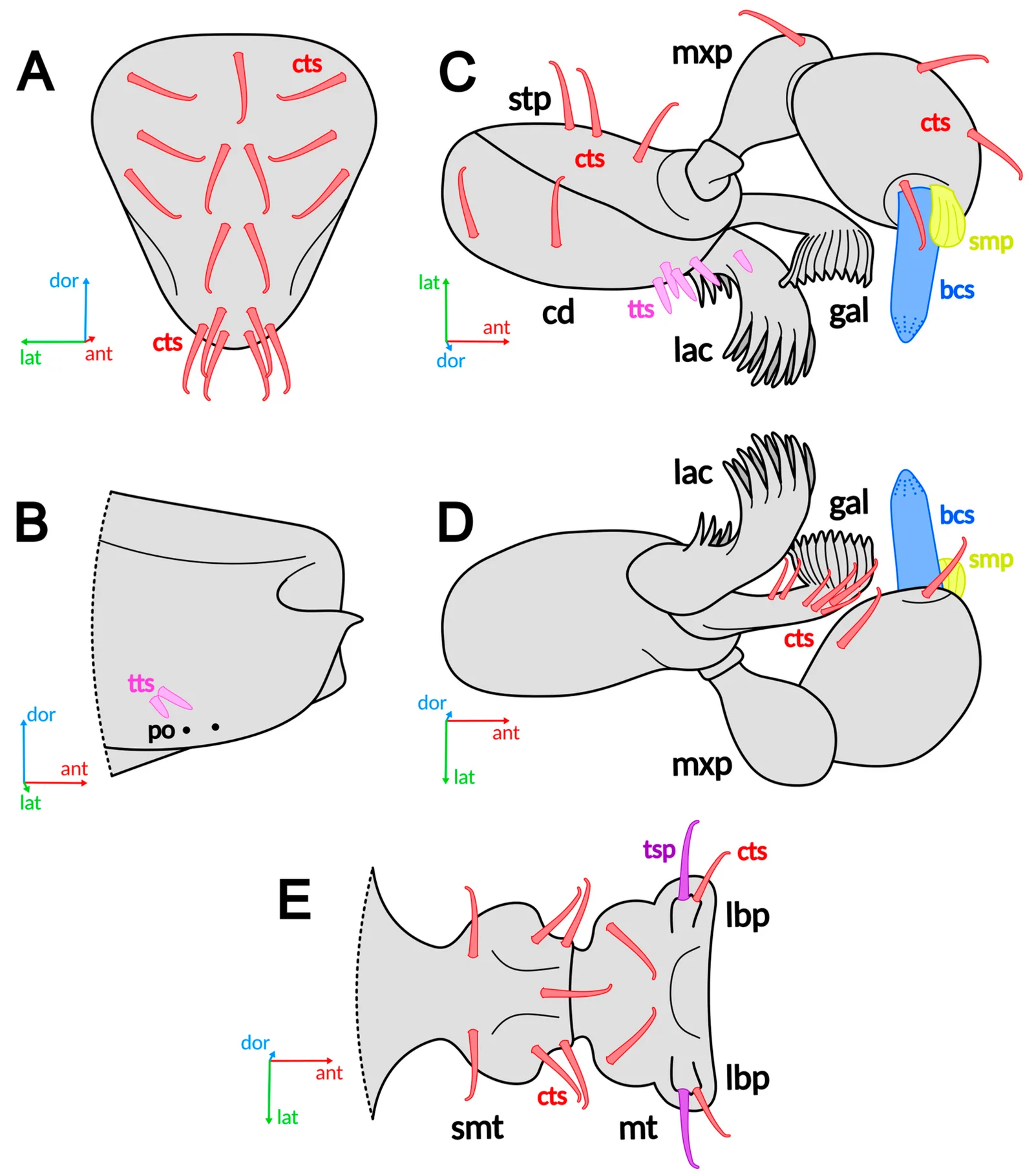
Distribution of sensilla on mouthparts of *S. musawasensis*. (**A**). Clypeus. (**B**). Mandible, lateral view. (**C**). Maxilla, ventro-lateral view. (**D**). Maxilla, dorso-medial view. (**E**). Labium, ventral view. Abbreviations: bcs—basiconic sensilla; cd—cardo; cts—curved trichoid sensilla; gal—galea; lac—lacinia; lbp—labial palp; mt—mentum; mxp—maxillary palp; po—pores; smp—structure on the third maxillary palpomere; smt—submentum; stp—stipes; tsp—porous trichoid sensilla; tsp—porous trichoid sensilla; tsp—porous trichoid sensilla; tts—thorn-like trichoid sensilla; ant—anterior direction; dor—dorsal direction; lat—lateral direction.

**Figure 5:**
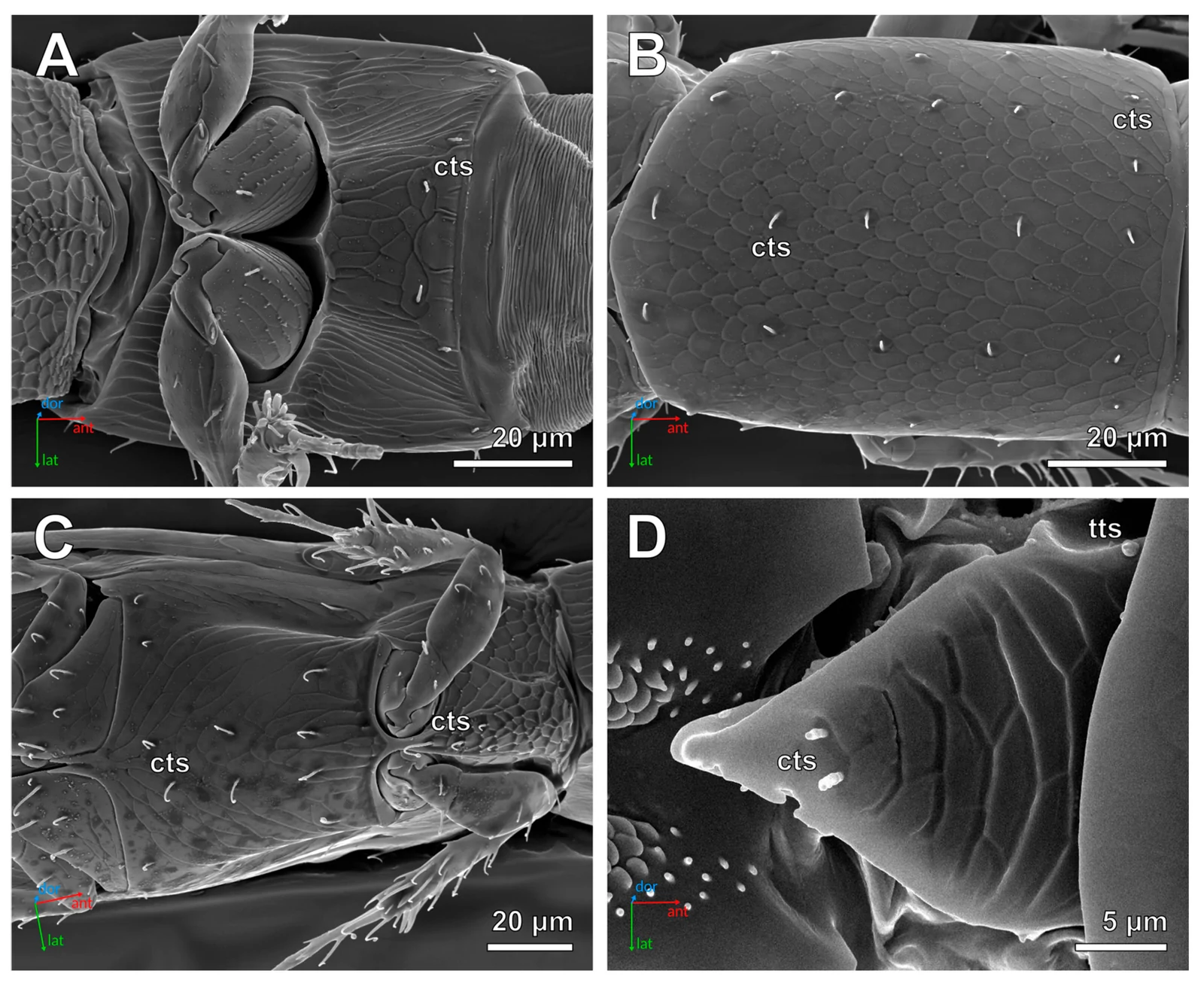
Thorax of *S. musawasensis* (SEM). (**A**). Ventral surface of prothorax, female. (**B**). Dorsal surface of prothorax, male. (**C**). Ventral surface of mesothorax and metathorax, male. (**D**). Scutellum, female. Abbreviations: cts—curved trichoid sensilla; tts—thorn-like trichoid sensilla; ant—anterior direction; dor—dorsal direction; lat—lateral direction.

**Figure 6:**
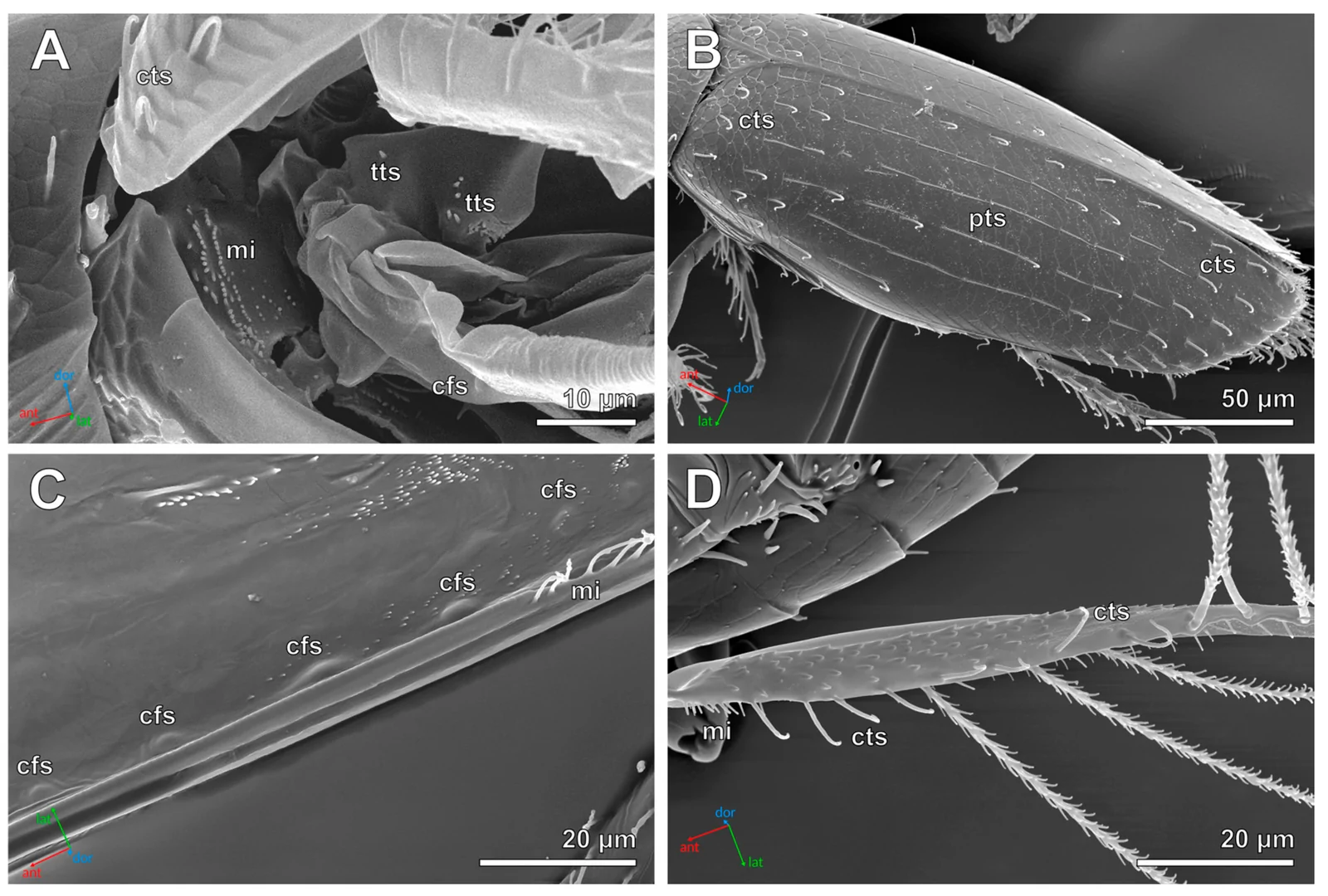
Elytra and hind wings of *S. musawasensis* (SEM). (**A**). Wing base, female. (**B**). Dorsal surface of elytron, male. (**C**). Row of campaniform sensilla on ventral surface of elytron, male. (**D**). Proximal part of hind wing, male. Abbreviations: cfs—campaniform sensilla; cts—curved trichoid sensilla; mi—microtrichia; pts—pointed trichoid sensilla; tts—thorn-like trichoid sensilla; ant—anterior direction; dor—dorsal direction; lat—lateral direction.

**Figure 7:**
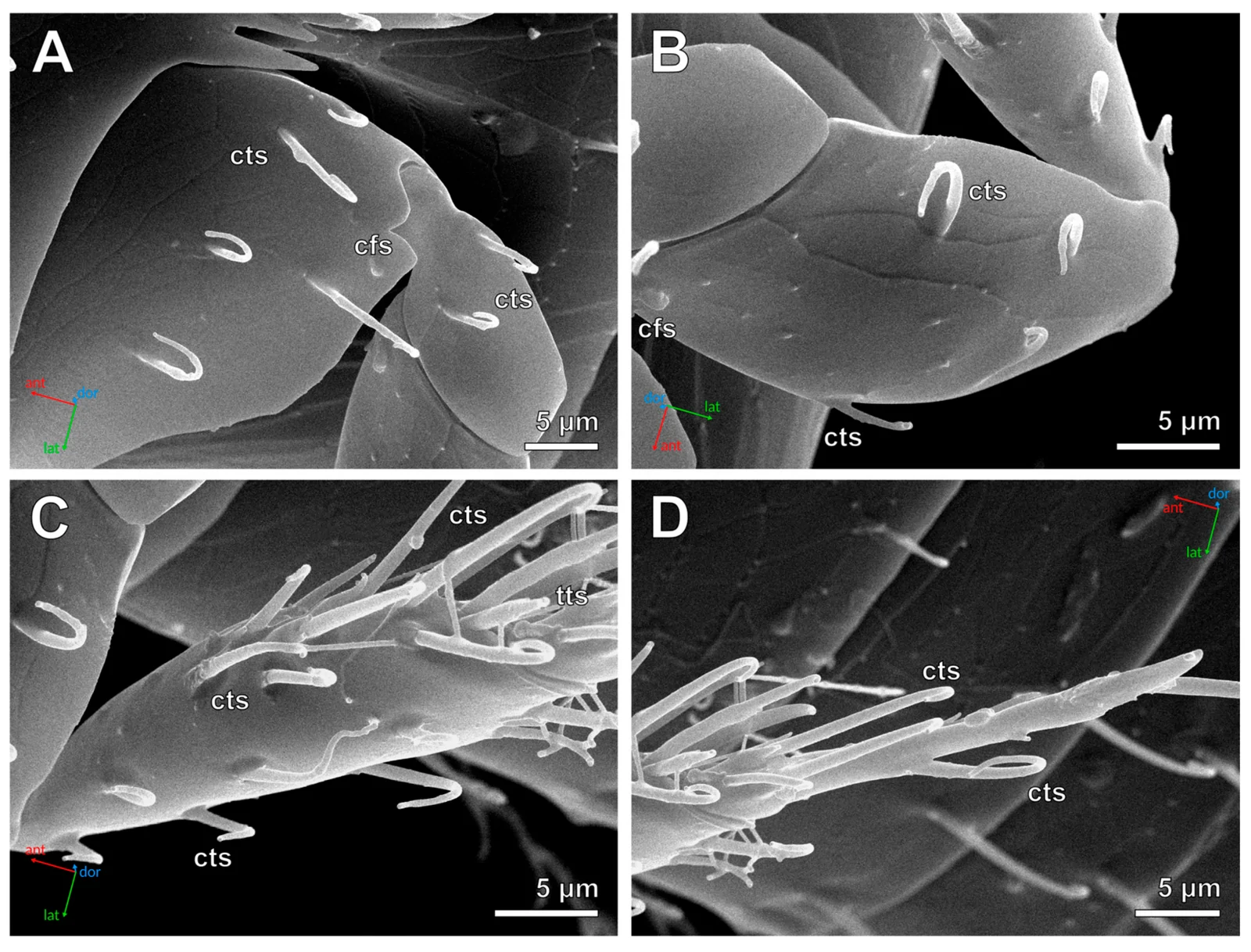
Hind leg of male *S. musawasensis*, ventral surface (SEM). (**A**). Coxa and trochanter. (**B**). Femur. (**C**). Tibia. (**D**). Tarsus. Abbreviations: cfs—campaniform sensilla; cts—curved trichoid sensilla; tts—thorn-like trichoid sensilla; ant—anterior direction; dor—dorsal direction; lat—lateral direction.

**Figure 8:**
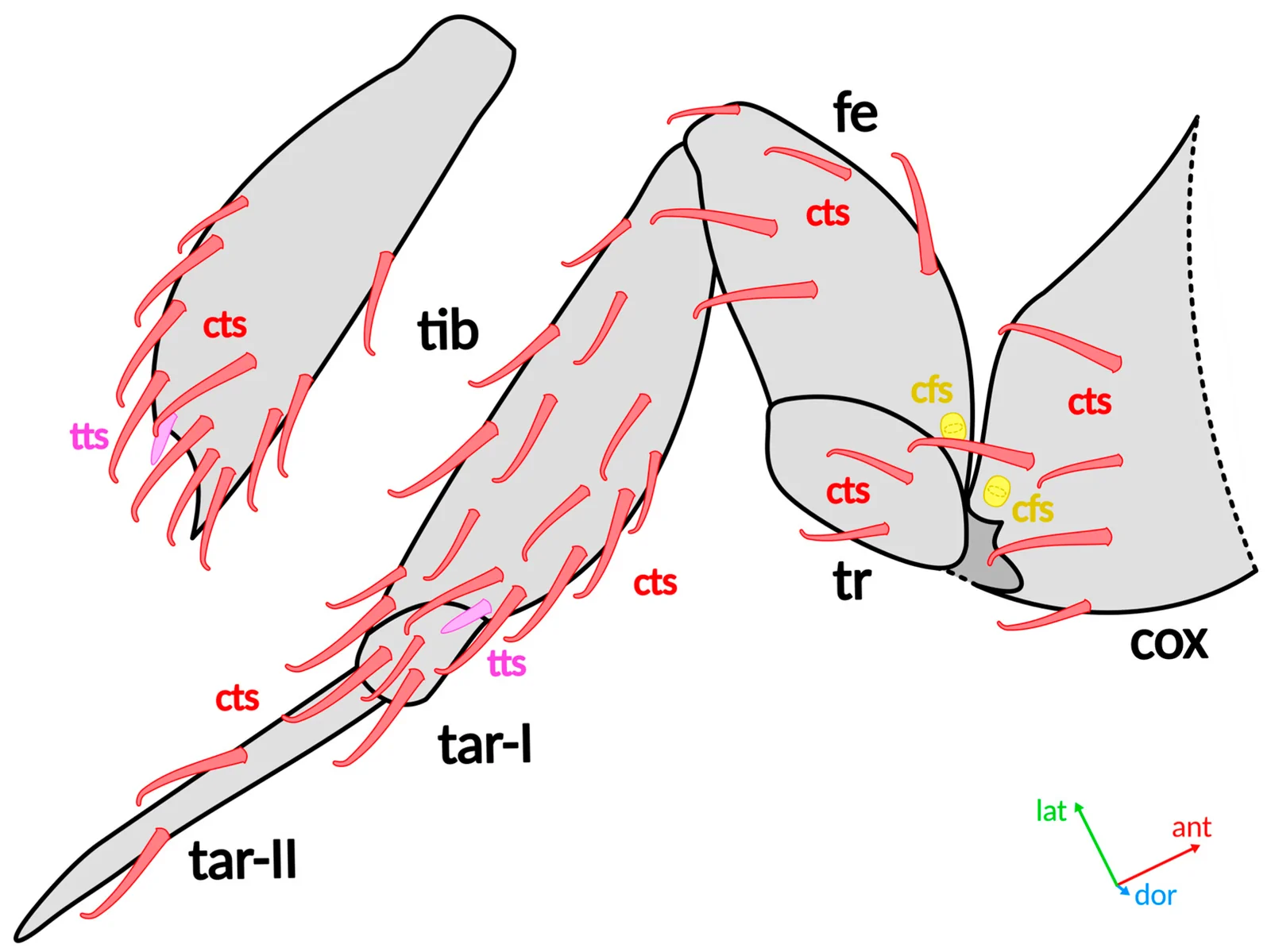
Distribution of sensilla on hind leg of *S. musawasensis*, ventral side. The dorsal side of the tibia is shown separately. Abbreviations: cfs—campaniform sensilla; cox—coxa; cts—curved trichoid sensilla; fe—femur; tar—tarsus; tib—tibia; tr—trochanter; tts—thorn-like trichoid sensilla; ant—anterior direction; dor—dorsal direction; lat—lateral direction.

**Figure 9:**
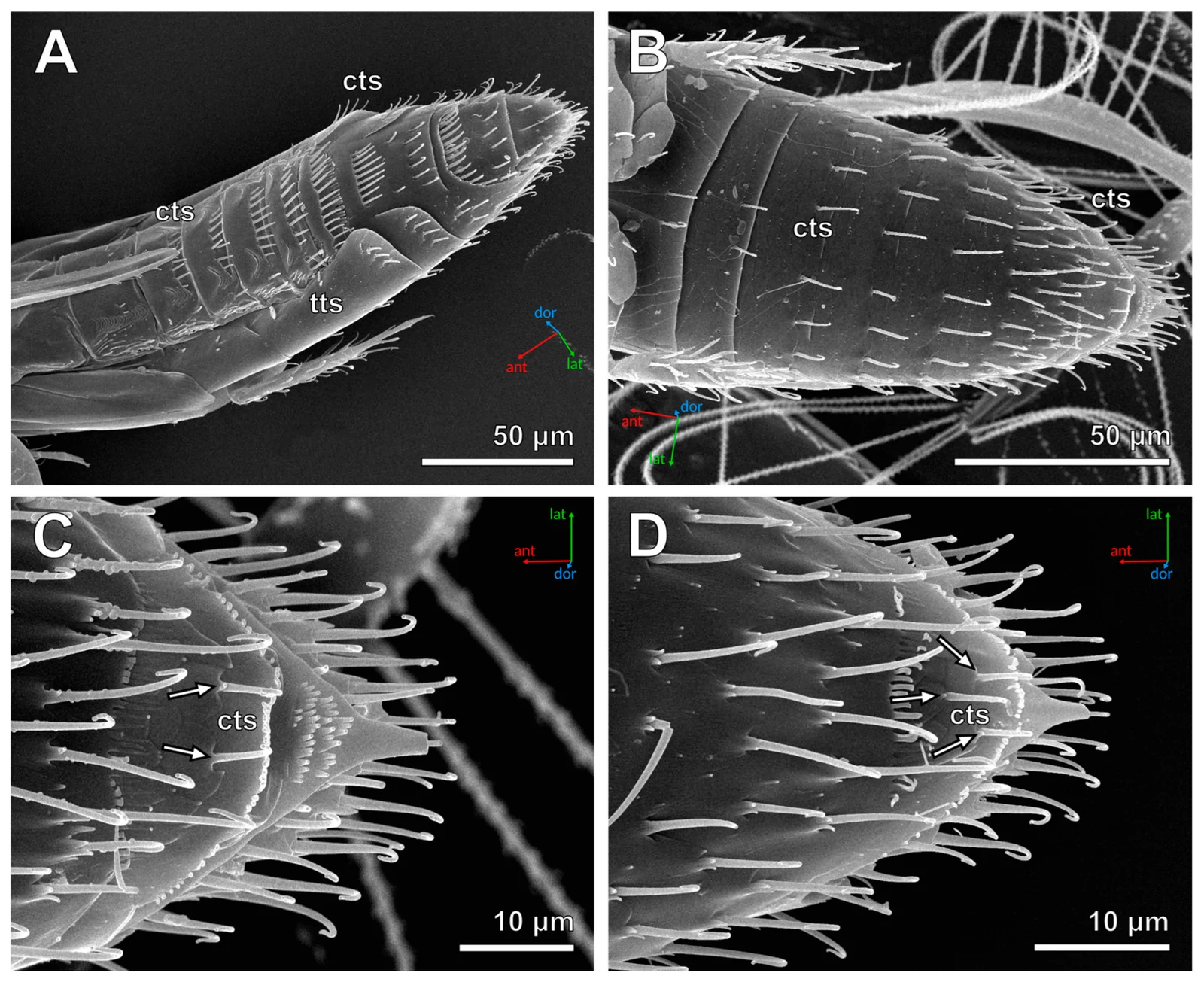
Abdomen of *S. musawasensis* (SEM). (**A**). Female abdomen, dorso-lateral view. (**B**). Male abdomen, ventral view. (**C**). Male pygidium, ventral view. (**D**). Female pygidium, ventral view. Arrows show different numbers of curved trichoid sensilla in males and females. Abbreviations: cts—curved trichoid sensilla; tts—thorn-like trichoid sensilla; ant—anterior direction; dor—dorsal direction; lat—lateral direction.

**Figure 10:**
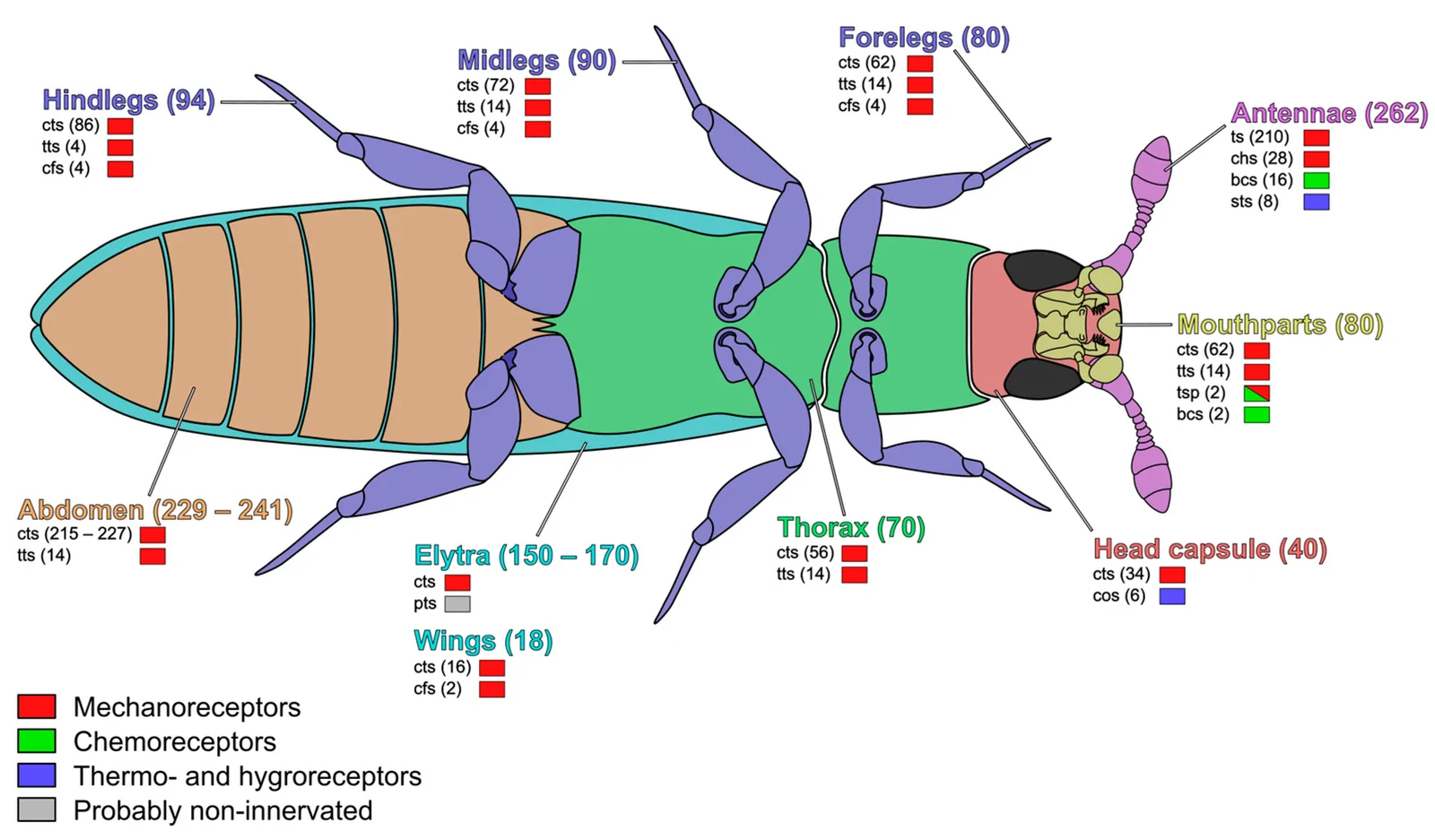
Distribution, number, and modality of sensilla types in *S. musawasensis*. Numbers of sensilla are given in brackets. Data on antennae are from Diakova and Polilov [28]. Abbreviations: bcs—basiconic sensilla; cfs—campaniform sensilla; chs—chaetoid sensilla; cos—coeloconic sensilla; cts—curved trichoid sensilla; pts—pointed trichoid sensilla; sts—styloconic sensilla; ts—trichoid sensilla; tsp—porous trichoid sensilla; tts—thorn-like trichoid sensilla.

## Data Availability

Data is contained within the article.

## Abbreviation

The following abbreviation is used in this manuscript:

SEM	Scanning electron microscopy
